# Emergent Behaviors from a Cellular Automaton Model for Invasive Tumor Growth in Heterogeneous Microenvironments

**DOI:** 10.1371/journal.pcbi.1002314

**Published:** 2011-12-22

**Authors:** Yang Jiao, Salvatore Torquato

**Affiliations:** 1 Physical Science in Oncology Center, Princeton Institute for the Science and Technology of Materials, Princeton University, Princeton, New Jersey, United States of America; 2 Department of Chemistry and Physics, Princeton Center for Theoretical Science, Program in Applied and Computational Mathematics, Princeton University, Princeton, New Jersey, United States of America; Johns Hopkins University, United States of America

## Abstract

Understanding tumor invasion and metastasis is of crucial importance for both fundamental cancer research and clinical practice. *In vitro* experiments have established that the invasive growth of malignant tumors is characterized by the dendritic invasive branches composed of chains of tumor cells emanating from the primary tumor mass. The preponderance of previous tumor simulations focused on non-invasive (or proliferative) growth. The formation of the invasive cell chains and their interactions with the primary tumor mass and host microenvironment are not well understood. Here, we present a novel cellular automaton (CA) model that enables one to efficiently simulate invasive tumor growth in a heterogeneous host microenvironment. By taking into account a variety of microscopic-scale tumor-host interactions, including the short-range mechanical interactions between tumor cells and tumor stroma, degradation of the extracellular matrix by the invasive cells and oxygen/nutrient gradient driven cell motions, our CA model predicts a rich spectrum of growth dynamics and emergent behaviors of invasive tumors. Besides robustly reproducing the salient features of dendritic invasive growth, such as least-resistance paths of cells and intrabranch homotype attraction, we also predict nontrivial coupling between the growth dynamics of the primary tumor mass and the invasive cells. In addition, we show that the properties of the host microenvironment can significantly affect tumor morphology and growth dynamics, emphasizing the importance of understanding the tumor-host interaction. The capability of our CA model suggests that sophisticated *in silico* tools could eventually be utilized in clinical situations to predict neoplastic progression and propose individualized optimal treatment strategies.

## Author Summary

The goal of the present work is to develop an efficient single-cell based cellular automaton (CA) model that enables one to investigate the growth dynamics and morphology of invasive solid tumors. Recent experiments have shown that highly malignant tumors develop dendritic branches composed of tumor cells that follow each other, which massively invade into the host microenvironment and ultimately lead to cancer metastasis. Previous theoretical/computational cancer modeling neither addressed the question of how such chain-like invasive branches form nor how they interact with the host microenvironment and the primary tumor. Our CA model, which incorporates a variety of microscopic-scale tumor-host interactions (e.g., the mechanical interactions between tumor cells and tumor stroma, degradation of the extracellular matrix by the tumor cells and oxygen/nutrient gradient driven cell motions), can robustly reproduce experimentally observed invasive tumor evolution and predict a wide spectrum of invasive tumor growth dynamics and emergent behaviors in various different heterogeneous environments. Further refinement of our CA model could eventually lead to the development of a powerful simulation tool for clinical purposes capable of predicting neoplastic progression and suggesting individualized optimal treatment strategies.

## Introduction

Cancer is not a single disease, but rather a highly complex and heterogeneous set of diseases that can adapt in an opportunistic manner, even under a variety of stresses. It is now well accepted that genome level changes in cells, resulting in the gain of function of oncoproteins or the loss of function of tumor suppressor proteins, initiate the transformation of normal cells into malignant ones and neoplastic progression [1], [2]. In the most aggressive form, malignant cells can leave the primary tumor, invade into surrounding tissues, find their way into the circulatory system (through vascular network) and be deposited at certain organs in the body, leading to the development of secondary tumors (i.e., metastases) [3].

**Figure 1:**
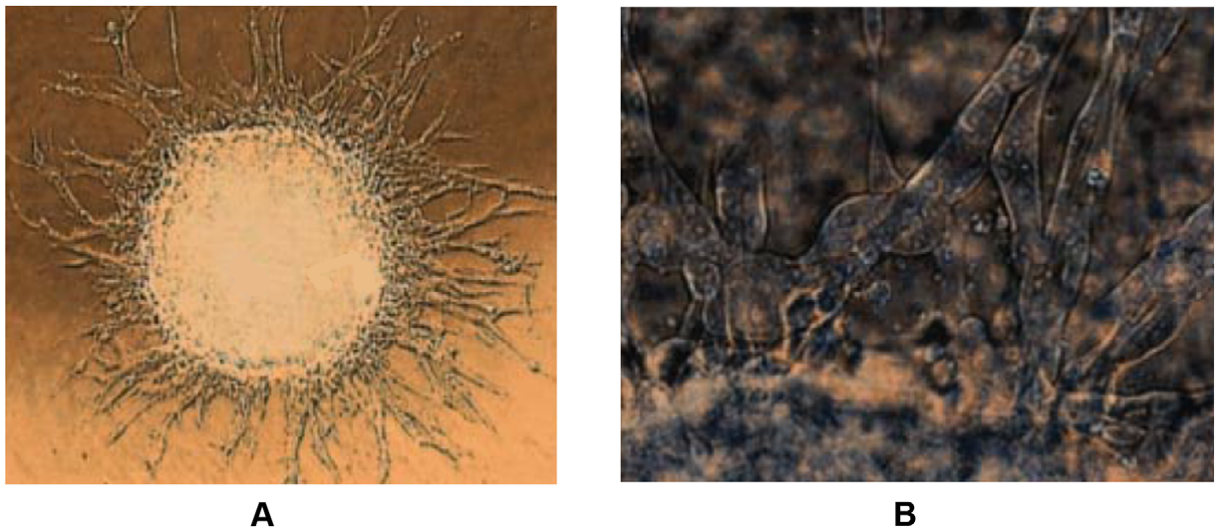
GBM multicelluar tumor spheroid (MTS) gel assay showing dendritic invasive branches. (a) The invasive branches centrifugal evolve from the central MTS. The linear size of central MTS is approximately 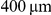. (b) The invasive branches are composed of chains of invasive cells. The images are adapted from Ref. [4].

The emergence of invasive behavior in cancer is fatal. For example, the malignant cells that invade into the surrounding host tissues can quickly adapt to various environmental stresses and develop resistance to therapies. The invasive cells that are left behind after resection are responsible for tumor recurrence and thus an ultimately fatal outcome. Therefore, significant effort has been expended to understand the mechanisms evolved in the invasive growth of malignant tumors [2], [4]–[7] and their treatment [8], [9]. It is generally accepted that the invasive behavior of cancer is the outcome of many complex interactions occurring between the tumor cells, and between a tumor and the host microenvironment [3]. Tumor invasion itself is a complex multistep process involving homotype detachment, enzymatic matrix degradation, integrin-mediated heterotype adhesion, as well as active, directed and random motility [4]. In recent *in vitro* experiments involving glioblastoma multiforme (GBM), the most malignant brain cancer, it has been observed that dendritic invading branches composed of chains of tumor cells are emanating from the primary tumor mass; see Figure 1. Such invasive behaviors are characterized by intrabranch homotype attraction and least-resistance paths of cells [4].

Although recent progress has been made in understanding certain aspects of the complex tumor-host interactions that may be responsible for invasive cancer behaviors [4], [10]–[12], many mechanisms are either not fully understood or are unknown at the moment. Even if all of the mechanisms for cancer invasion could be identified, it is still not clear that progress in understanding neoplastic progression and proposing individualized optimal treatment strategies could be made without the knowledge of how these different mechanisms couple to one another and to the heterogeneous host microenvironment in which tumor grows [13]. Theoretical/computational cancer modeling that integrates distinctly different mechanisms for tumorigenesis, when appropriately linked with experimental and clinical data, offers a promising avenue for a better understanding of tumor growth, invasion and metastasis. A successful model would enable one to broaden the conclusions drawn from existing medical data, suggest new experiments, test hypotheses, predict behavior in experimentally unobservable situations, and be employed for early detection and prognosis [13].

Indeed, cancer modeling has been a very active area of research for the last two decades (see Refs. [13] and [14] for recent reviews). A variety of interactions between the tumor and its host microenvironment have been investigated [15]–[32] via continuum [25]–[28], [32], discrete [16], [20], [33] or hybrid [19], [21]–[23] mathematical models. Very recently, multiscale mathematical models [22], [23], [28] have been employed to study the effects of the host microenvironment on the morphology and phenotypic evolution of invasive tumors and it has been shown that microenvironmental heterogeneity can dramatically affect the growth dynamics of invasive tumors. Although these simulated tumors predicted certain invasive characteristics (e.g., development of protruding surfaces), no dendritic invasive branches emerged from these numerical studies.

In response to the challenge to develop an “Ising” model for cancer growth [13], we generalize here a cell-based discrete cellular automaton (CA) model that we have developed [16]–[18], [20], [21] to investigate the invasive growth of malignant tumors in heterogeneous host microenvironments. To the best of our knowledge, this generalized CA model is the first to investigate the formation of invasive cell chains and their interactions with the primary tumor mass and host microenvironment. Our generalized cellular automaton model takes into account a variety of microscopic-scale tumor-host interactions, including the short-range mechanical interactions between tumor cells and tumor stroma, the degradation of extracellular matrix by the invasive cells and oxygen/nutrient gradient driven cell motions and thus, it can predict a wide range of growth dynamics and emergent behaviors of invasive tumors. In particular, our CA model robustly reproduces the salient features of dendritic invasive growth observed in experiments, which is characterized by least-resistance paths of cells and intrabranch homotype attraction. The model also predicts nontrivial coupling between the growth dynamics of the primary tumor mass and the invasive cells, e.g., the invasive cells can facilitate the growth of primary tumor in harsh microenvironment. Moreover, we show that the properties of the host microenvironment can significantly affect tumor growth dynamics and lead to a variety of tumor morphologies. These emergent behaviors naturally arise due to various microscopic-scale tumor-host interactions, which emphasizes the importance of taking into account microenvironmental heterogeneity in understanding cancer. Further refinement of our model could eventually lead to the development of a powerful *in silico* tool that could be utilized in the clinic. As a demonstration of the capability and versatility of our CA model, we mainly consider invasive tumor growth in two dimensions, although the model is easily extended to three dimensions. Indeed, the algorithmic details of the model are given for any spatial dimension.

## Materials and Methods

### Biophysical Background of the CA Model

#### Voronoi Tessellation: the underlying cellular structure

**Figure 2:**
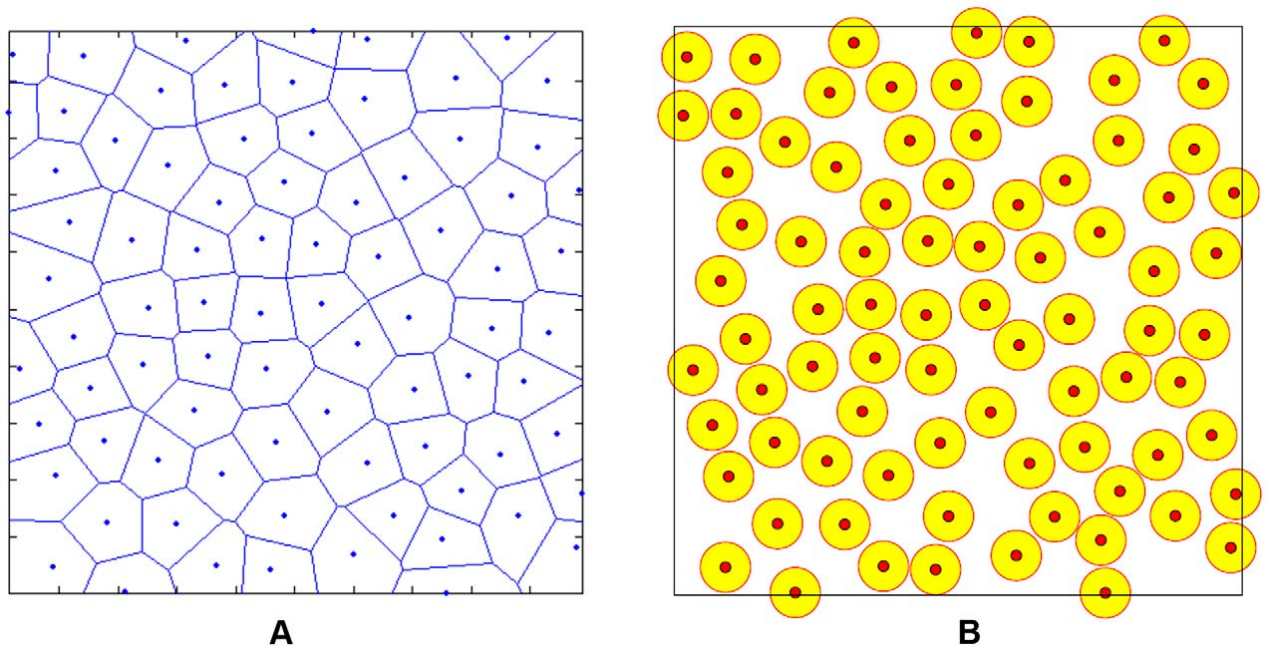
A 2D Voronoi tessellation and the associated point configuration. (a) A Voronoi tessellation of the 2D plane into polygons which are the automaton cells in our model. (b) The associated point configuration for the tessellation, generated by randomly placing nonoverlap circular disks in a prescribed region, i.e., the random sequential addition process [34].

The underlying cellular structure is modeled using a Voronoi tessellation of the space into polyhedra [34], based on centers of spheres in a packing generated by a random sequential addition (RSA) process [16], [21] (see Figure 2). In particular, nonoverlapping spheres are randomly and sequentially placed in a prescribed region until there is no void space left for additional spheres, i.e., *saturation* is achieved. Such a saturated RSA packing possesses relative small variations in its Voronoi polyhedra and thus, has served as models for many biological systems [35], [36]. We refer to the polyhedra associated with the Voronoi tessellation as automaton cells. These automaton cells may correspond to real biological cells or tumor stroma (e.g., clusters of the ECM macromolecules). In previous studies, such automaton cells have represented clusters of real cells of various sizes or have implicitly represented healthy tissues [16]. Thus, the Voronoi tessellation associated with RSA sphere centers provides a highly flexible model for real-cell aggregates with a relatively high degree of shape isotropy. For example, one can use a variable automaton cell size to simulate avascular tumor growth from a few malignant cells to its macroscopic size [16]. In addition, such a Voronoi tessellation can reduce the undesired growth bias due to the anisotropy of ordered tessellations based on square and simple cubic lattices in two and three dimensions, respectively.

Since our new CA model explicitly takes into account the interactions between a single biological cell and its neighbors and microenvironment, each automaton cell here represents either a single tumor cell or a region of tumor stroma. Thus, the linear size of a single automaton cell is approximately 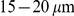 and the linear size of the 2D simulation domain is approximately 5 mm, which contains 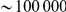 automaton cells. In the current model, we mainly focus on the effects of the ECM macromolecule density and ECM degradation by malignant cells on tumor growth. Henceforth, we will refer to the host microenvironment (or tumor stroma) as the “ECM” for simplicity. Each ECM associated automaton cell is assigned a particular density 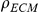, representing the density of the ECM macromolecules within the automaton cell. A tumor cell can occupy an ECM associated automaton cell only if the density of this automaton cell 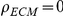, which means that either the ECM is degraded or it is deformed (pushed away) by the proliferating tumor cells.

#### Microenvironment heterogeneity

The microenvironment in which tumor grows is usually highly heterogeneous, composed of various types of stromal cells and ECM structures. The ECM is a complex mixture of macromolecules that provides mechanical support for the tissue (such as collagen) and those that play an important role for cell adhesion and motility (such as laminin and fibronectin) [22], [37], [38]. For different individuals with tumors, the ECM in the host microenvironments generally possess distinct mechanical and transport properties. By explicitly representing the ECM using automaton cells with different macromolecule densities, the effects of microenvironment heterogeneity on tumor growth can be very well explored. For example, various distributions of the ECM densities (i.e., the densities of the ECM macromolecules) can be employed to mimic the actual heterogeneous host microenvironment of the tumor. Certain tumor stroma contain fibroblasts, which actively produce ECM macromolecules leading to a higher ECM density around these cells. The automaton cells representing the ECM with larger densities are considered to be more rigid and more difficult to degrade. Since each automaton cell associated with the ECM has its own density, this allows a variation of the ECM characteristics on the length scale comparable to that of a single tumor cell.

In addition, the tumor in our model is only allowed to grow in a compact growth-permitting region. This is done to mimic the physical confinement of the host microenvironment, such as the boundary of an organ. In other words, only automaton cells within this region can be occupied by the cells of the tumor as it grows. In general, the growth-permitting region can be of any shape that best models the organ shape. Here we simply choose a spherical region to study the effects of the heterogeneous ECM on tumor growth. More sophisticated growth-region shapes have been employed to investigate the effects of physical confinement on tumor growth [20], [21]. Furthermore, we assume a constant radially symmetric nutrient/oxygen gradient in the growth-permitting region with the highest nutrition concentration at the boundary of this region, i.e., it is a vascular boundary. However, this assumption can also be relaxed.

#### Tumor cell phenotypes and interactions with the host microenvironment

For highly malignant tumors, we consider the cells to be of one of the two classes of phenotypes: either invasive or non-invasive. Following Ref. [16], the non-invasive cells remain in the primary tumor and can be proliferative, quiescent or necrotic, depending on the nutrient supply they get. For avascular tumor growth, our focus here, the nutrients available to the tumor cells are essentially the nutrient concentrations that diffuse into the tumor through its surface. As the tumor grows, the amount of nutrient supply, which is proportional to the surface area of the tumor, cannot meet the needs of all cells whose number increases with the tumor volume, leading to the development of necrotic and quiescent regions. Following Ref. [16], characteristic diffusion lengths are employed to determine the states of a non-invasive cell. For example, quiescent cells more than 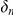 away from the tumor surface become necrotic (see details in the next section). The diffusion length 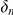 (also the characteristic thickness of the rim of living tumor cells) depends on the size of the primary tumor.

As a proliferative cell divides, its daughter cell effectively pushes away/degrades the surrounding ECM and occupies the automaton cell originally associated with the ECM [39]–[41]. It is easier for a tumor cell to take up an ECM associated automaton cell with lower density (i.e., less rigid ECM regions) than that with higher density (i.e., more rigid ECM regions) and thus, the tumor growth is affected by the ECM heterogeneity through the local mechanical interaction between tumor cells and the ECM. If there is no space available for the placement of a daughter cell within a distance 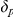 from the proliferative cell, the proliferative cell turns quiescent.

The invasive cells are considered to be mutant daughters of the proliferative cells [42], which can gain a variety of degrees of the ECM degradation ability 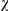 (i.e., the matrix-degradation enzymes) and motility 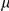 that allow them to leave the primary tumor and invade into surrounding microenvironment [43]. We consider that the invasive cells can move from one automaton cell to another only if the ECM in the target automaton cell is completely degraded (i.e., with 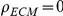). Each trial move of an invasive cell involves the degradation of the ECM in its neighbor automaton cells, followed by a possible move to one of the automaton cells whose ECM is completely degraded; otherwise the invasive cell does not move. The number of trial moves of an invasive cell and to what extent it degrades the ECM are respectively determined by 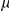 and 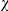 (see the following section for details). The oxygen/nutrient gradient also drives the invasive cells to move as far as possible from the primary tumor [44], which takes up the majority of oxygen/nutrients. The motility 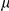 is the maximum possible number of such trial moves. In addition, we assume that the invasive cells do not divide as they migrate.

### Algorithmic Details

We now provide specific details for the CA model to study invasive tumor growth in confined heterogeneous microenvironment. In what follows, we will simply refer to the primary tumor as “the tumor” and explicitly use “invasive” when considering invasive cells. After generating the automaton cells by Voronoi tessellation of RSA sphere centers, an ECM macromolecule density 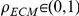 is assigned to each automaton cell within the growth-permitting region, which represents the heterogeneous host microenvironment. Then a tumor is introduced by designating any one or more of the automaton cells as proliferative cancer cells. Time is then discretized into units that represent one real day. At each time step:

- Each automaton cell is checked for type: invasive, proliferative, quiescent, necrotic or ECM associated. Invasive cells degrade and migrate into the ECM surrounding the tumor. Proliferative cells are actively dividing cancer cells, quiescent cancer cells are those that are alive, but do not have enough oxygen and nutrients to support cellular division and necrotic cells are dead cancer cells.
- All ECM associated automaton cells and tumorous necrotic cells are inert (i.e., they do not change type).
- Quiescent cells more than a certain distance 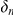 from the tumor's edge are turned necrotic. The tumor's edge, which is assumed to be the source of oxygen and nutrients, consists of all ECM associated automaton cells that border the neoplasm. The critical distance 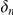 for quiescent cells to turn necrotic is computed as follows:
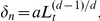
where 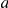 is prescribed parameter (see Table 1), 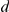 is the spatial dimension and 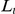 is the distance between the geometric centroid (i.e., the center) 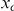 of the tumor and the tumor edge cell that is closest to the quiescent cell under consideration. The position of the tumor centroid 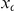 is given by
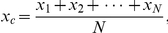
where 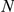 is the total number of noninvasive cells contained in the tumor, which is updated when a new noninvasive daughter cell is added to the tumor.
- Each proliferative cell will attempt to divide with probability 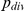 into the surrounding ECM (i.e., the automaton cells associated with the ECM) by degrading and pushing away the ECM in that automaton cell. We consider that 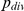 depends on both the physical confinement imposed by the boundary of the growth-permitting region and the local mechanical interaction between the tumor cells and the ECM, i.e.,
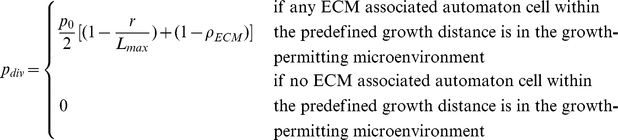
where 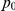 is the base probability of division (see Table 1), 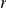 is the distance of the dividing cell from the tumor centroid, 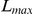 is the distance between the closest growth-permitting boundary cell in the direction of tumor growth and the tumor's geometric centroid 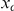 and 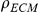 is the ECM density of the automaton cell to be taken by the new tumor cell. When an ECM associated automaton cell is taken by a tumor cell, its density is set to be zero. The predefined growth distance (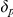) is described in a following bullet point.
- If a proliferative cell divides, it can produce a mutant daughter cell possessing an invasive phenotype with a prescribed probability 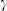 (i.e., the mutation rate). The invasive daughter cell gains ECM degradation ability 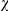 and motility 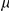, which enable it to leave the tumor and invade into the surrounding ECM. The rules for updating invasive cells are given in a following bullet point. If the daughter cell is noninvasive, it is designated as a new proliferative cell.
- A proliferative cell turns quiescent if there is no space available for the placement of a daughter cell within a distance 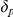 from the proliferative cell, which is given by
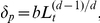
where 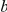 a nutritional parameter (see Table 1), 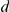 is the spatial dimension and 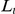 is the distance between the geometric tumor centroid 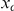 and the tumor edge cell that is closest to the proliferative cell under consideration.
- An invasive cell degrades the surrounding ECM (i.e., those in the neighboring automaton cells of the invasive cell) and can move from one automaton cell to another if the associated ECM in that automaton cell is completely degraded. For an invasive cell with motility 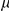 and ECM degradation ability 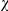, it will make 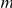 attempts to degrade the ECM in the neighboring automaton cells and jump to these automaton cells, where 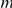 is an arbitrary integer in 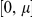. For each attempt, the surrounding ECM density 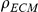 is decreased by 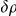, where 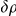 is an arbitrary number in 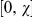. Using random numbers for ECM degradation ability and cellular motility is to take into account tumor genome heterogeneity, which is manifested as heterogeneous phenotypes (such as different 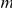 and 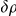). When the ECM in multiple neighboring automaton cells of the invasive cell are completely degraded (i.e., 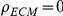), the invasive cell moves in a direction that maximizes the nutrient and oxygen supply. Here we assume that the migrating invasive cells do not divide. The degraded ECM shows the invasive path of the tumor.

**Table 1:** Parameters and terms in the CA model.

**Time dependent terms**	
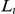	Local tumor radius (varies with cell positions)
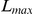	Local maximum tumor extent (varies with cell positions)
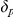	Characteristic proliferative rim thickness
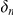	Characteristic living-cell rim thickness (determines necrotic fraction)
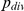	Probability of division (varies with cell positions)
**Growth parameters**	
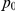	Base probability of division, linked to cell-doubling time (0.192 and 0.384)
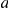	Base necrotic thickness, controlled by nutritional needs ( 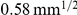 )
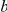	Base proliferative thickness, controlled by nutritional needs ( 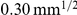 )
**Invasiveness parameters**	
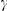	Mutation rate (determines the number of invasive cells, 0.05)
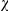	ECM degradation ability ( 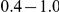 )
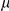	Cell motility (the number of “jumps” from one automaton cell to another, 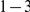 )
**Other terms**	
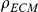	ECM density (determines the ECM rigidity and varies with positions, 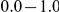 )

Summarized here are definitions of the parameters for tumor growth and invasion, and all other (time-dependent) quantities used in the simulations. For each parameter, the number(s) listed in parentheses indicates the value or range of values assigned to the parameters during the simulations. The values of the parameters are chosen such that the CA model can reproduce reported growth dynamics of GBM from the medical literature [4], [16], [20].

**Figure 3:**
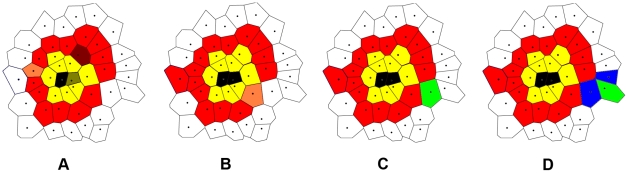
Illustration of cellular automaton rules. Necrotic cells are black, quiescent cells are yellow, proliferative cells are red and invasive tumor cells are green. The ECM associated automaton cells are white and the degraded ECM is blue. (a) A proliferative cell (dark red) is too far away from the tumor edge to get sufficient nutrients/oxygen and it will turn quiescent in panel (b). A quiescent cell (dark yellow) is too far away from the tumor edge and it will turn necrotic in panel (b). Another proliferative cell (light red) will produce a daughter proliferative cell in panel (b). (b) The dark red proliferative cell and the dark yellow quiescent cell in panel (a) turned quiescent and necrotic, respectively. The light red proliferative cell in panel (a) produced a daughter cell. Another proliferative cell (light red) will produce a mutant invasive daughter cell. (c) The light red proliferative cell in (b) produced an invasive cell. (d) The invasive cell degraded the surrounding ECM and moved to another automaton cell.

The aforementioned automaton rules are briefly illustrated in Figure 3. We note that non-invasive tumor growth can be studied by imposing a mutation rate 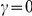. This enables us to compare the growth dynamics of invasive and non-invasive tumors and in turn to investigate the effects of the coupling between the growth dynamics of the primary tumor mass and the invasive cells. Although we only consider spherical growth-permitting regions here, the CA rules given above allow growth-permitting regions with arbitrary shapes. The important parameters mentioned in the bullet points above are summarized in Table 1. In the following, we will employ our CA model to investigate the growth dynamics of malignant tumors with different degrees of invasiveness in a variety of different heterogeneous microenvironments.

### Quantitative Metrics for Tumor Morphology

To characterize quantitatively the morphology of simulated tumors, we present several scalar metrics that capture the salient geometric features of the primary tumor, dendritic invasive branches or the entire invasive pattern. These metrics include the ratio 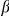 of the invasive area over the tumor area (defined below), the specific surface 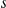 of the invasive pattern, the asphericity 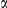 of the primary tumor and the angular anisotropy metric 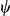 for the invasive branches. The metrics are computed for all simulated tumors and compared to available experimental data. We note that the invasive pattern associated with a neoplasm includes both the primary tumor and the invasive branches.

**Figure 4:**
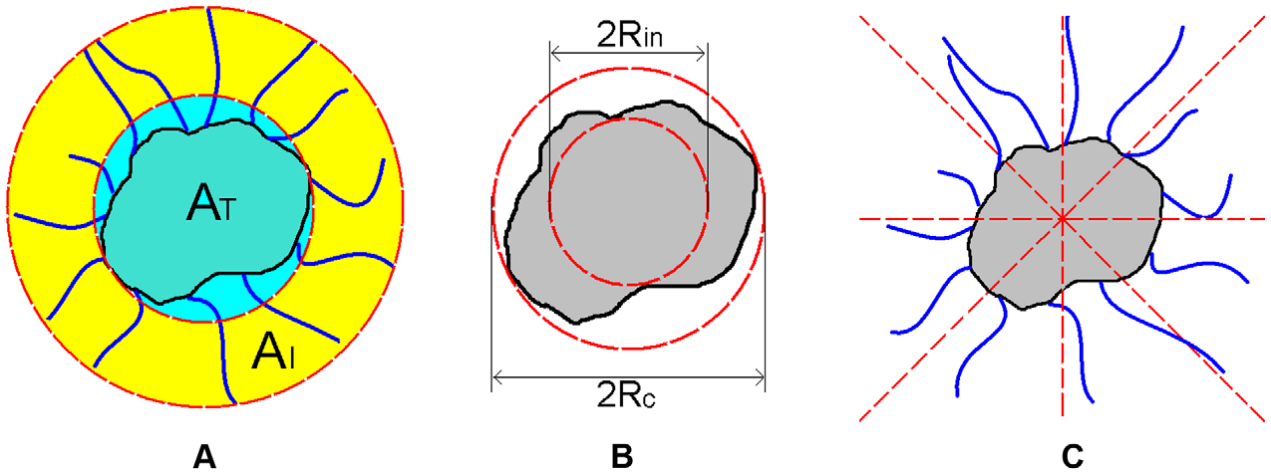
Schematic illustration of the quantities in the definitions of tumor morphology metrics. (a) Invasive area 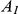 and tumor area 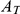 associated with the invasive pattern. (b) Circumcircle with radius 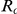 and incircle with radius 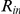 associated with the primary tumor. (c) Evenly dividing the invasive pattern into 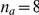 sectors for computing the angular anisotropy metric 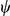.

Following Ref. [4], the tumor area 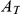 is defined as the area of the circumcircle of the primary tumor (see Figure 4(a)) and the invasive area 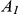 is the area of the region between the effective circumcircle of the invasive pattern and the circumcircle of the primary tumor (see Figure 4(a)). The radius of the effective circumcircle of the invasive pattern is defined to be the average distance from the invasive branch tip to the tumor center. The ratio 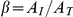 as a function of time 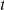 reflects the degree of coupling between the primary tumor and the invasive cells. If 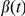 is linear in 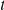, there is no coupling; otherwise the two are coupled.

The specific surface 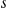 [34] for the invasive pattern is defined as the ratio of the total length of the perimeter of the invasive pattern over its total area. In general, 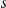 is inversely proportional to the size of the tumor and thus, large tumors have small 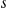 values. Moreover, given the tumor size, tumors with a large number of long dendritic invasive branches possess a large value of 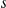. And 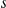 is minimized for perfectly circular tumors with 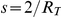, where 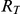 is the radius. Since 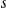 depends on the size of the tumor, which makes it difficult to compare tumors with different sizes, in the calculations that follow we employ a normalized 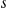 with respect to 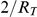 for an arbitrary-shaped tumor with effective radius 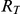 (i.e., the average distance from tumor edge to tumor center). For simplicity, we will still refer to the normalized specific surface as “specific surface” and designate it with symbol 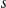.

The asphericity 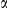 of the primary tumor is defined as the ratio of the radius of circumcircle 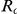 of the primary tumor over its incircle radius 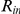 [45], i.e., 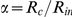 (see Figure 4(b)). A large 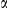 value indicates a large deviation of the shape of primary tumor from that of a perfect circle, i.e., the tumor is more anisotropic.

To quantify the degree of anisotropy of the invasive branches, we introduce the angular anisotropy metric 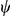. In particular, the entire invasive pattern is evenly divided into 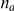 sectors with lines emanating from the tumor center (see Figure 4(c)). The angular anisotropy metric 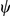 is defined as
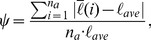
(1)where 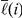 is the average length of the invasive branches within the 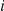
th sector and
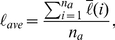
(2)is the average length of all invasive branches. For tumors with invasive branches of similar lengths that are uniformly angularly distributed, the metric 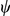 is small. Large fluctuations of both invasive branch length and angular distribution can lead to large 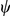 values. In the following, we use 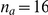 to compute 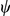 for the simulated invasive tumors.

## Results

### Model Validation

To verify the robustness and predictive capacity of our CA model, we first employ it to reproduce quantitatively the observed invasive growth of a GBM multicellular tumor spheroid (MTS) *in vitro* [4]. In particular, the boundary of the growth-permitting region is considered to be vascularized, i.e., a growing tumor can receive oxygen and nutrients from the growth-permitting region. A constant radially symmetric nutrient/oxygen gradient in the growth-permitting region with the highest nutrient/oxygen concentration at the vascular boundary is used. Initially, approximately 250 proliferative tumor cells are introduced at the center of the growth-permitting region with homogeneous ECM and tumor growth is started. This corresponds to an initial MTS with diameter 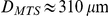 which is consistent with the *in vitro* experiment set-up [4]. The following values of the growth and invasiveness parameters are used: 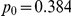, 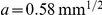, 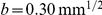, 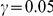, 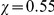, 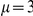. Note that the value of 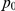 corresponds to a cell doubling time of 40 hours, which is consistent with the reported experimental data [4]. A small value of the ECM density 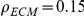 is used, which corresponds to the soft DMEM medium used in the experiment [4]. In the visualizations of the tumor that follow, we use the following convention: the ECM in the growth-permitting region is white, and gray outside this region. The ECM degraded by the tumor cells is blue. In the primary tumor, necrotic cells are black, quiescent cells are yellow and proliferative cells are red. The invasive tumor cells are green.

**Figure 5:**
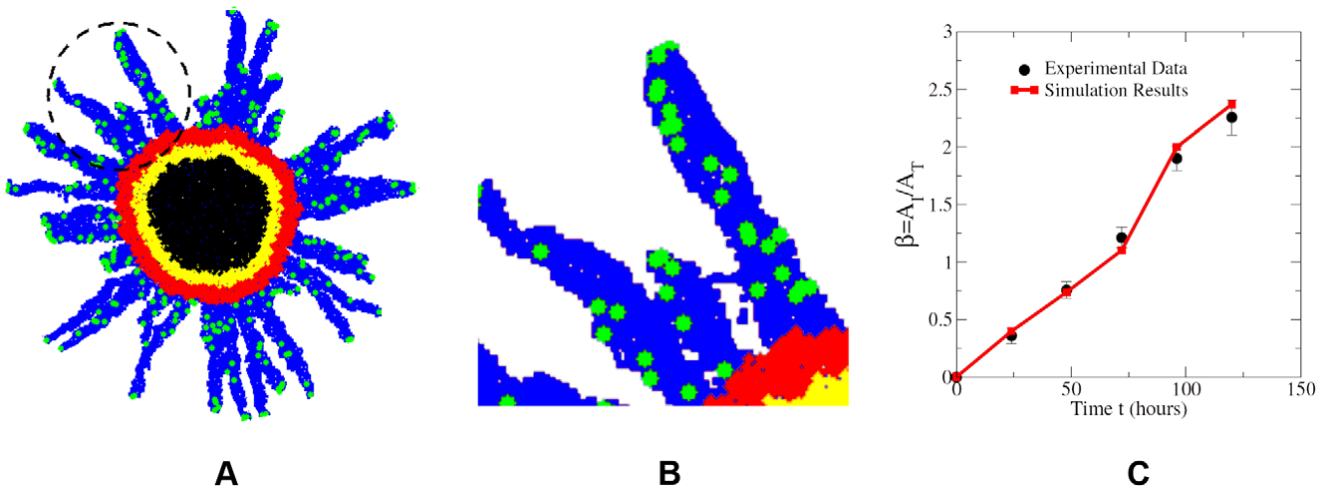
Simulated invasive growth of MTS *in vitro*. (a) A snapshot of the simulated growing MTS at 24 hours after initialization. The region circled is magnified in panel (b). (b) A magnification of the circled region in panel (a). One can clearly see that the invasive cells (green) are following each other to form chains within the dendritic branches (blue), as observed in experiment [4]. (c) Comparison of 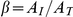 as a function of time associated with the simulated MTS and the *in vitro* experimental data [4].

Figure 5(a) and (b) respectively show the morphology of simulated MTS and a magnification of its invasive branches with increasing branch width towards the proliferative core. Specifically, one can clearly see that within the branches, chains of cells are formed as observed in experiments [4] (see Figure 1). The invasive cells tend to follow one another (which is termed “homotype attraction”) since paths of degraded ECM are formed by pioneering invasive cells and it is easier for other cells to follow and enhance such paths than degrading ECM to create new paths by themselves. In other words, invasive cells tend to take paths with “least resistance”. We note that no CA rules are imposed to force such cellular behaviors. Instead, they are emergent properties that arise in our simulations.

The ratio of the invasion area over the primary tumor area 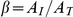 as a function of time for the simulated tumor is computed and compared to the reported experimental data [4] (see Figure 5(c)). One can clearly see that our simulation results agree with experimental data very well. Moreover, the deviation of 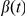 from a linear function of 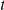 indicates that the growth of primary tumor and the invasive branches are strongly coupled [4]. Other metrics for tumor morphology such as the specific surface 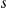 of the invasive pattern, the sphericity 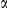 of the primary tumor and the angular anisotropy metric 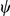 for the invasive branches are computed from our simulation results and from the image of invasive MTS in Figure 1(a) at 24 hours after initialization. The values are given in Table 2, from which one can see again a good agreement. Thus, we have shown that our CA model is both robust and quantitatively accurate with properly selected parameters.

**Table 2:** Comparison of tumor morphology metrics associated with simulated MTS and experimental data at 24 hours after tumor initialization.

Metrics	Simulated MTS	Experimental data
Specific surface 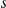	9.24	9.78
Asphericity 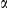	1.09	1.12
Angular anisotropy metric 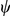	0.17	0.19

### Simulated Invasive Growth in Heterogeneous Miroenvironments

**Figure 6:**
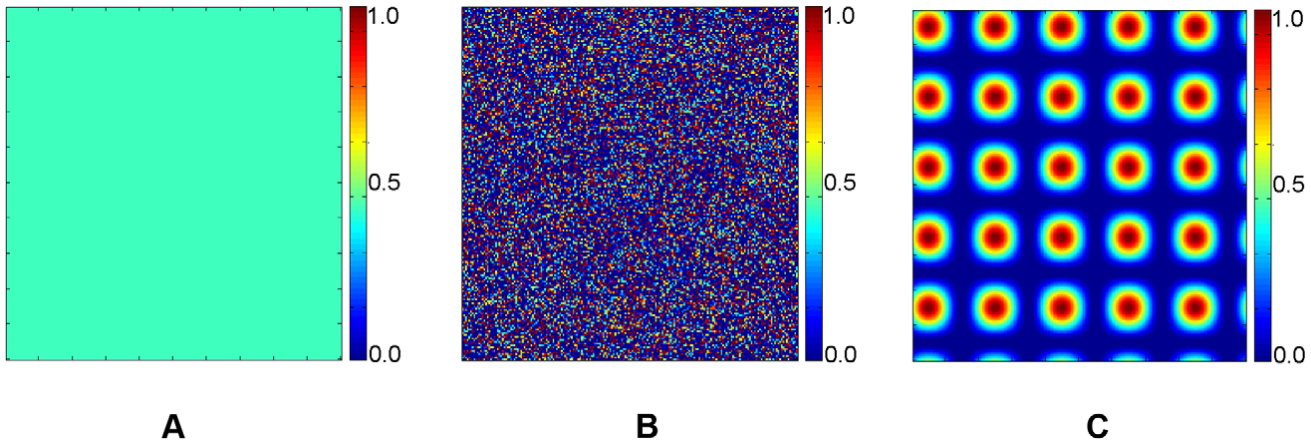
Different distributions of the ECM densities. (a) Uniform distribution. (b) Random distribution, i.e., the value of 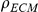 is completely independent of 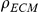 values of other automaton cells. (c) Sinusoidal-like ECM density distribution defined by Eq. (3) to mimic the obstacles for a growing tumor.

Having verified the robustness and predictive capacity of our CA model, we now consider three types of distributions of the ECM density, i.e., homogeneous, random and sinusoidal-like, to systematically study the effects of microenvironment heterogeneity on invasive tumor growth (see Figure 6). These ECM density distributions represent real host microenvironments in which a tumor grows. (Details about these ECM distributions are given in the following sections.) Again, the boundary of the growth-permitting region is considered to be vascularized with a constant radially symmetric nutrient/oxygen gradient in the growth-permitting region pointing to the tumor center. We note that although generally the nutrient/oxygen concentration field *in vivo* is more complicated than exhibited here, previous numerical studies that considered the exact evolution of nutrient/oxygen concentrations have shown a decay of the concentrations toward the tumor center [22], [23]. Since the directions of cell motions are determined by the nutrient/oxygen gradient, our constant-gradient approximation is a very reasonable one.

In the beginning of the simulation, a proliferative tumor cell is introduced at the center of the growth-permitting region and tumor growth is initiated. The growth parameters for the primary tumors in all cases studied here are the same and are given in Table 1. The invasiveness parameters and ECM densities are variables and specified in each case separately. The values of the growth parameters for the CA model were chosen to be consistent with GBM data from the medical literature [16], [20]. Specifically, the value of the base probability of division is 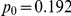, which corresponds to a cell doubling time of 4 days [46], [47]. This value is used for all of the cases of invasive growth that follow. Since our CA model takes into account general microscopic tumor-host interactions, we expect that the general growth dynamics and emergent behaviors predicted by the model will qualitatively apply to other solid tumors. We note that all of the reported growth dynamics and emergent properties of the simulated tumors for any specific set of growth and invasiveness parameters are repeatedly observed in 25 independent simulations.

#### Effects of cellular motility

We first simulate the growth of malignant tumors with different degrees of invasiveness in a homogeneous ECM with 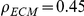. In particular, we consider three invasive cases with the same mutation rate 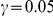 and ECM degradation ability 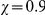, but different cell motility 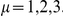 A non-invasive growth case (i.e., 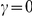) in the same microenvironment (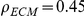) is also studied for comparison purposes.

**Figure 7:**
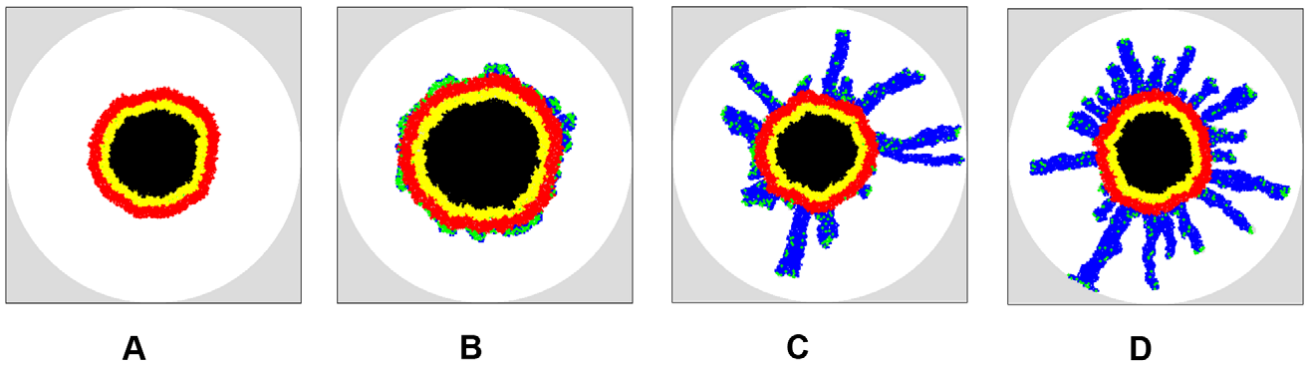
Simulated growing tumors in homogeneous ECM with 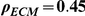 on day 100 after initiation. For the invasive growth, the mutation rate is 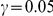 and ECM degradation ability is 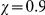, (a) Tumor cells are noninvasive, i.e., 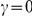. (b) Invasive tumor with cellular motility 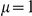. (c) Invasive tumor with cellular motility 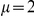. (d) Invasive tumor with cellular motility 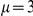. Note that the size of the primary tumor whose growth is facilitated by the concentric-like shell formed by clumpped invasive cells (b) is much larger than the other cases. Invasive cells with a larger motility lead to more dendritic invasive branches. Although we only consider spherical growth-permitting regions here, our tumor-growth simulations can incorporate growth-permitting regions with arbitrary shapes.

Figure 7 shows the simulated growing tumors 100 days after initiation (plots showing the full growth history of the tumors are given in Figure S1, S2, S3, S4). The computed metrics for tumor morphology are given in Table 3. The primary tumors for both invasive (Figure 7(b),(c) and (d)) and non-invasive (Figure 7(a)) cases develop necrotic and quiescent regions. For invasive tumors, when the cell motility is small (i.e., 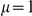), the invasive cells do not form dendritic invasive branches but rather clump near the outer border of the proliferative rim (see Figure 7(b)), forming bumpy invasive concentric-like shells with relatively small specific surface (e.g., 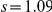 on day 100). Such invasive shells significantly enhance the growth the primary tumor, i.e., the size of the primary tumor in Figure 7(b) is much larger than in Figure 7(a), (c) and (d). A quantitative comparison of the tumor sizes is shown in Figure S5.

**Table 3:** Morphology metrics for simulated tumors growing in homogeneous ECM.

Noninvasive tumor in ECM with 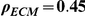				
Metrics	Day 50	Day 80	Day 100	Day 120
Specific surface 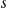	1.23	1.13	1.09	1.04
Asphericity 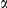	1.21	1.18	1.08	1.12

By contrast, for larger cell motility, long dendritic invasive branches are developed as manifested by the large specific surface (e.g., 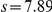 on day 100 and 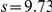 on day 120 for 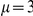). In particular, one can clearly see that within the branches the cells tend to follow one another to form chains, as observed in experiments [4]. We emphasize that no rules are imposed to force the cells to follow one another in our CA model. This homotype attraction is purely due to the mechanical interaction between the invasive cells and the ECM, i.e., once a path of invasion is established by a pioneering invasive cell (by degrading the ECM), other invasive cells nearby turn to follow and enhance this path since the resistance for migration is minimized on a existing path. Furthermore, we can see that larger cell motility (i.e., high malignancy) leads to more invasive branches (see Figure 7(c) and (d)) and thus, a larger specific area of the invasive pattern.

#### Effects of the ECM rigidity

It is not very surprising that isotropic tumor shapes and invasive patterns are developed in a homogeneous ECM with relative low density (i.e., the ECM is soft) compared to the ECM degradation ability of the invasive tumor cells. However, real tumors are rarely isotropic, primarily due to the host microenvironment in which they grow, which we now explore.

**Figure 8:**
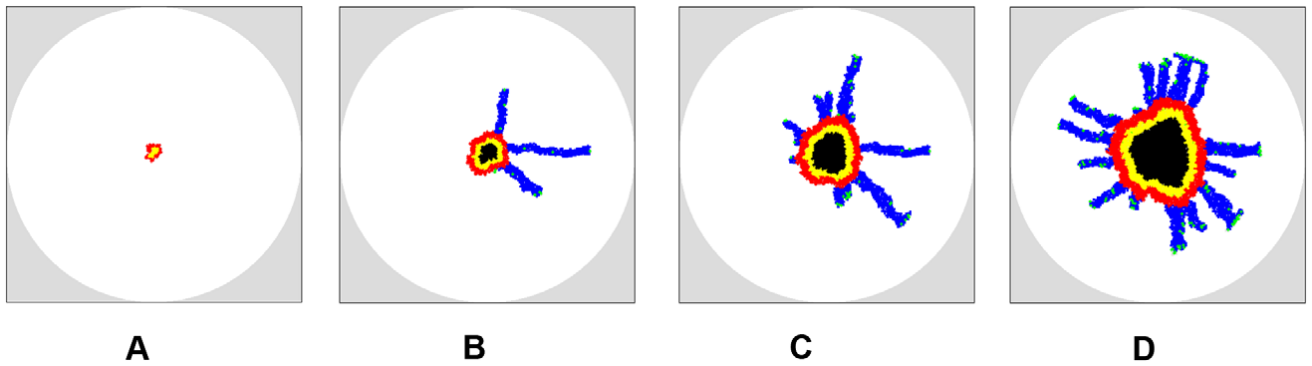
Evolution of simulated tumor in homogeneous ECM with 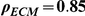. The mutation rate is 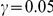, the cell motility is 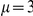 and ECM degradation ability is 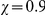. (a) Growing tumor on day 50. (b) Growing tumor on day 80. (c) Growing tumor on day 100. (d) Growing tumor on day 120. Note that although the ECM is homogeneous, due to its high rigidity, the primary tumor develops an anisotropic shape with protrusions in the proliferation rim caused by the invasive branches. Also note that the invasive cells clump at the tips of certain invasive branches due to the high ECM rigidity. Although we only consider spherical growth-permitting regions here, our tumor-growth simulations can incorporate growth-permitting regions with arbitrary shapes.

Consider the invasive growth of a tumor in a much more rigid ECM than that in the previous section, i.e., 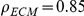. The invasiveness parameters used are 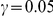, 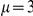 and 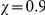. The snap shots of the growing tumor are shown in Figure 8 and the tumor morphology metrics are given in Table 3. It can be clearly seen that both the size of the primary tumor and the extent of its invasive branches are much smaller than those of the tumors growing in a softer ECM (see Figure 7). Importantly, although the ECM is still homogeneous, due to its high rigidity, the primary tumor develops an anisotropic shape with protrusions in the proliferation rim caused by the invasive branches (e.g., 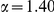 and 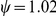 on day 100). Since the invasive cells have degraded the ECM either completely or partially along the invasive branches, it is easier for the proliferative cells in the primary tumor to take these “weak spots” than to push against the rigid ECM themselves. Again, we emphasize that we do not force the cells to behave this way by imposing special CA rules; this behavior results purely from the mechanical interaction between the tumor and its host and the coupling between the growth dynamics of the invasive and non-invasive tumor cells.

#### Effects of the ECM heterogeneity: random distribution of the ECM density

**Figure 9:**
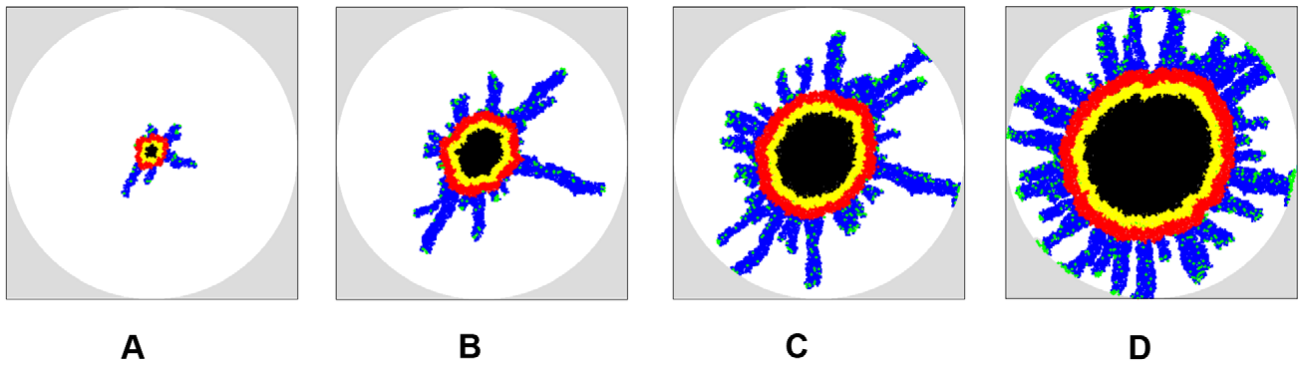
Evolution of simulated tumor in random ECM. The mutation rate is 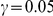, the cell motility is 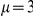 and ECM degradation ability is 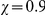. (a) Growing tumor on day 50. (b) Growing tumor on day 80. (c) Growing tumor on day 100. (d) Growing tumor on day 120. Note that both the primary tumor and invasive pattern are affected (i.e., becoming anisotropic) by the ECM heterogeniety in the early growth stages. Also note that unlike the case in Figure 6(d), the invasive cells clump at the tips of invasive branches since they have reached the boundary of the growth-permitting region. Although we only consider spherical growth-permitting regions here, our tumor-growth simulations can incorporate growth-permitting regions with arbitrary shapes.

The real host microenvironment for tumors are far from homogeneous in general. To investigate how ECM heterogeneity affects the tumor growth dynamics, we use a random distribution of the ECM density, i.e., for each ECM associated automaton cell, its density 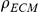 is a random number uniformly chosen from the interval 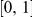 (see Figure 6(b)). The invasiveness parameters used are 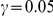, 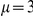 and 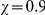 and the snap shots of the growing tumor are shown in Figure 9. The tumor morphology metrics are given in Table 4. Note that the primary tumor develops a rough surface and slightly anisotropic shape in the early growth stages (e.g., 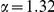 on day 50 and 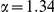 on day 80), which reflects the ECM heterogeneity (Figure 9(a) and (b)). Since the characteristic heterogeneity length scale is comparable to a single cell, its effects are diminished (e.g., 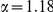 on day 100 and 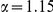 on day 120) as the tumor grows larger and larger (Figure 9(c) and (d)). (In other words, on large length scales, the ECM is still effectively homogeneous.) However, the anisotropy in the invasive pattern (i.e., the extents of invasive branches in different directions) still persists (e.g., 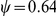 on day 100) even though the primary tumors almost resumes an isotropic shape.

**Table 4:** Morphology metrics for simulated tumors growing in heterogeneous ECM.

Invasive tumor with 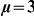 in random ECM				
Metrics	Day 50	Day 80	Day 100	Day 120
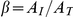	2.54	4.14	2.13	2.78
Specific surface 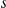	2.47	3.97	4.65	8.98
Asphericity 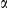	1.32	1.34	1.18	1.15
Angular anisotropy metric 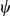	0.63	0.87	0.64	0.19

#### Effects of the ECM heterogeneity: sinusoidal-like distribution of the ECM density

To represent large-scale heterogeneities in the ECM, we use a sinusoidal-like distribution of the ECM density, i.e., for an automaton cell with centroid 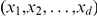, the associated ECM density is given by
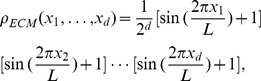
(3)where 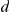 is the spatial dimension and 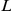 is the edge length of the 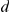-dimensional cubic simulation box. A two-dimensional sinusoidal-like ECM density distribution is shown in Figure 6(c). The red spots correspond to large 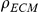 and high ECM rigidity; they can be considered as effective obstacles (e.g., brain ventricles) that hinder tumor growth.

**Figure 10:**
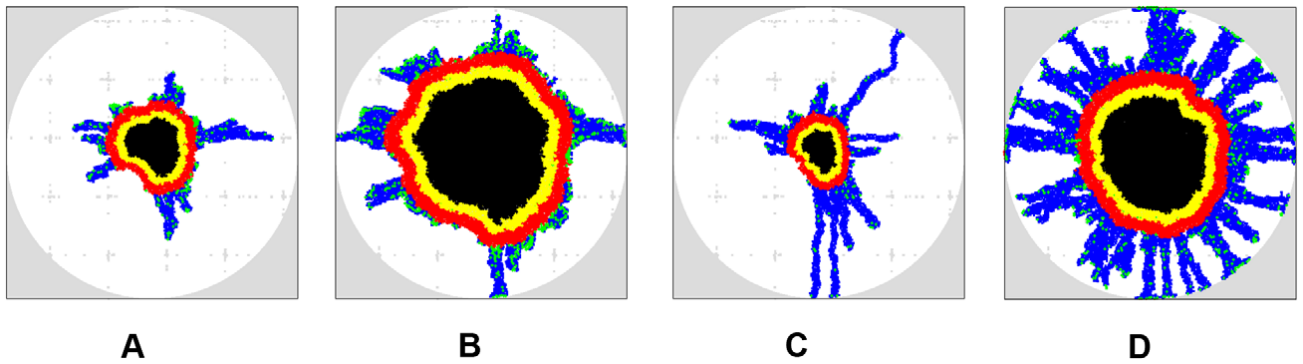
Simulated growing tumors in the ECM having a sinusoidal-like density distribution with different cellular motilities (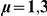). The mutation rate is 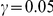 and ECM degradation ability is 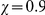. (a) Growing tumor with cell motility 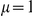 on day 80. (b) Growing tumor with cell motility 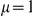 on day 120. (c) Growing tumor with cell motility 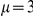 on day 80. (d) Growing tumor with cell motility 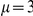 on day 120. Note that both the primary tumor and invasive pattern in the two cases are significantly affected by the ECM heterogeneity, i.e., the tumors are highly anisotropic in shape and the invasive branches clearly favor two orthogonal directions associated with low ECM densities in the early growth stages. Although we only consider spherical growth-permitting regions here, our tumor-growth simulations can incorporate growth-permitting regions with arbitrary shapes.

Figures 10(a),(b) and (c),(d) show the snap shots of invasive tumors growing in the aforementioned ECM on day 80 and day 120, with invasiveness parameters 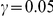, 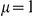 and 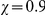 and 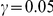, 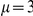 and 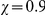, respectively. The plots showing the full growth history is given in Figure S6 and S7, and the tumor morphology metrics are given in Table 4. We can see that in the early growth stage, both the primary tumor and invasive pattern in the two cases are significantly affected by the ECM heterogeneity. In particular, the tumors are highly anisotropic in shape and the invasive branches clearly favor two orthogonal directions associated with low ECM densities (e.g., 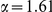, 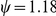 for 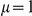 on day 80; and 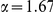, 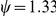 for 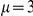 on day 80). For the case with large cellular motility, anisotropy effects are diminished in later growth stages (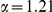, 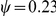 for 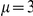 on day 120). For small cellular motility, anisotropy in both primary tumor shape and the invasive pattern persists (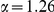, 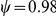 for 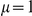 on day 120). Furthermore, one can see that again invasive cells with low motility significantly facilitate the growth of the primary tumor. However, instead of forming “bumpy” concentric-like shells as in homogeneous ECM, the invasive cells form large invasive cones, protruding into the ECM. These invasive cones are followed by weak protrusion of the proliferative rim, leading to bumpy surface of the primary tumor. The fact that such complex growth dynamics are only observed for tumors growing heterogeneous ECM emphasizes the crucial importance of understanding the effects of physical heterogeneity in cancer research.

## Discussion

We have developed a novel cellular automaton (CA) model which, with just a few parameters, can produce a rich spectrum of growth dynamics for invasive tumors in heterogeneous host microenvironment. Besides robustly reproducing the salient features of branched invasive growth, such as least-resistance paths of cells and intrabranch homotype attraction observed in *in vitro* experiments, our model also enables us to systematically investigate the effects of microenvironment heterogeneity on tumor growth as well as the coupling between the growth dynamics of the primary tumor and the invasive cells. In particular, we have shown that in homogeneous ECM with low densities (i.e., soft microenvironment), both the shape of the primary tumor and invasive pattern are isotropic. For high cellular motility cases, the invasive cells form extended dendritic invasive branches; while for low cellular motility cases, the invasive cells clump near the primary tumor surface and form a bumpy concentric-like shell that facilitates the growth of the primary tumor. Tumors growing in a highly rigid homogeneous ECM can develop anisotropic shapes, facilitated by the invasive cells that degrade the ECM; both the tumor size and the extent of invasive branches are much smaller. In heterogeneous ECM, both the primary tumor and invasive pattern are significantly affected during the early growth stages, i.e., anisotropic shapes and patterns are developed to avoid high density/rigid regions of the ECM. If the characteristic length scale of the heterogeneities is comparable to the macroscopic tumor size, such effects can persist in later growth stages. In addition, invasive cells with large motility can significantly diminish the anisotropy effects by their ECM degradation activities. We emphasize that we did not manipulate the behavior of cells by imposing artificial CA rules to give rise to these complex and rich growth dynamics. Instead, these are emergent behaviors that naturally arise due to various microscopic-scale tumor-host interactions that are incorporated into our CA model, including the short-range mechanical interaction between the tumor cells and tumor stroma, and the degradation of extracellular matrix by the invasive cells.

It is noteworthy that the growth dynamics of tumors in a heterogeneous microenvironment is distinctly different than those in a homogeneous microenvironment. This emphasizes the importance of understanding the effects of physical heterogeneity of the host microenvironment in modeling tumor growth. Here we just make a first attempt to take into account a simple level of host heterogeneity, i.e., by considering the ECM with variable density/rigidity. Currently, the invasion of the malignant cells into the host microenivronment is considered to be a consequence of invasive cell phenotype gained by mutation, and is not triggered by environmental stresses. However, the effects of environmental stresses can be taken into account. For example, a CA rule can be imposed that if the division probability of a malignant cell is significantly reduced by ECM rigidity, i.e., it is extremely difficult to push away/degrade ECM to make room for daughter cells, the malignant cell leaves the primary tumor and invades into soft regions of surrounding ECM. This would lead to reduced tumor invasion (i.e., development of the dendritic invasive branches) in soft microenvironments but enhanced invasion in rigid microenvironments [48]. Indeed, we have very recently generalized the CA model reported here to explicitly take into account the pressure exerted on the tumor due to the deformation of its surrounding ECM as well as the local geometry of the tumor-host interface to study mechanical-stress induced tumor morphology instability [49].

Moreover, the spatial-temporal evolution of more complicated and realistic nutrient/oxygen fields can be incorporated into our CA model. This can be achieved by solving the coupled nonlinear partial differential equations governing the evolution of the nutrient/oxygen concentrations as was done in Refs. [19] and [21]. Since the CA rules are given for any spatial dimension, our model is readily generalized to three dimensions. In addition, the model can be modified to incorporate other host heterogeneities, such as stromal cells, blood vessels and the shape anisotropy of the host organ [20], [21]. As currently implemented, a single 2D simulation takes less than 0.5 hours on a 32-bit 1.56 Gb Memory 1.44 GHz dual core Dell Workstation. We expect that a 3D simulation will take no longer than 24 hours on a supercomputer when a proper parallel implementation is used.

Such an *in silico* tool not only enables one to investigate tumor growth in complex heterogeneous microenvironment that closely represents the real host microenvironments but also allows one to infer and even reconstruct individual host microenvironment given limited growth data of tumors (such as shape and size at various times). Such microstructural information of the individual host would be extremely valuable for developing individualized treatment strategies. For example, based on the host microstructure one can design special encapsulation and transport agents that maximize drug delivery efficiency [13].

In our current CA model, the microscopic parameters governing tumor invasion are variable and can be arbitrarily chosen within a feasible range as given in Table 1. Given sufficient and reliable experimental data of invasive tumor growth, the parameters in our CA model could be uniquely determined and thus, the model could produce robust predictions about neoplastic progression. Although the current CA model is specifically implemented to reproduce and predict the growth dynamics of invasive solid tumors *in vitro*, further refinement of the model could eventually lead to the development of a powerful simulation tool that could some day be utilized clinically. For example, more complicated and realistic host heterogeneities such as the vascular structure, various stromal cells, the corresponding spatial-temporal evolution of the nutrient/oxygen concentrations as well as environmental stress-induced mutations should be incorporated as we described earlier. If the robustness of the refined model could be validated clinically, we would expect it to produce quantitative predictions for *in vivo* tumor growth, which could potentially be valuable for tumor prognosis and proposing individualized treatment strategies.

## Supporting Information

Figure S1 **Evolution of a simulated non-invasive (proliferative) tumor in a homogeneous ECM.** (a) Growing tumor on day 50. (b) Growing tumor on day 80. (c) Growing tumor on day 100. (d) Growing tumor on day 120. Note that the tumor morphology on day 100 is shown in Figure 5(a) of the main paper.
(TIF)

Figure S2
**Evolution of a simulated invasive tumor with cellular motility** 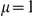 **in a homogeneous ECM.** The mutation rate is 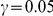 and ECM degradation ability is 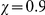. (a) Growing tumor on day 50. (b) Growing tumor on day 80. (c) Growing tumor on day 100. (d) Growing tumor on day 120. Note that the tumor morphology on day 100 is shown in Figure 5(b) of the main paper.(TIF)

Figure S3
**Evolution of a simulated invasive tumor with cellular motility** 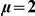 **in a homogeneous ECM.** The mutation rate is 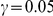 and ECM degradation ability is 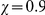. (a) Growing tumor on day 50. (b) Growing tumor on day 80. (c) Growing tumor on day 100. (d) Growing tumor on day 120. Note that the tumor morphology on day 100 is shown in Figure 5(c) of the main paper.(TIF)

Figure S4
**Evolution of a simulated invasive tumor with cellular motility** 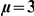 **in a homogeneous ECM.** The mutation rate is 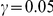 and ECM degradation ability is 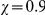. (a) Growing tumor on day 50. (b) Growing tumor on day 80. (c) Growing tumor on day 100. (d) Growing tumor on day 120. Note that the tumor morphology on day 100 is shown in Figure 5(d) of the main paper.(TIF)

Figure S5
**The scaled volume** 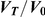 **of the primary tumors as function of time** 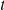 **.** The volume of the tumor 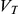 is scaled with respect to the volume of the growth-permitting region 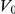. Note that invasive cells with small motility 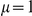 significantly enhance the growth of the primary tumor. For invasive cells with intermediate (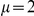) and large (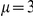) motility values, since such invaisve cells leave the primary tumor and do not contribute to the tumor volume, the size of primary tumors are smaller than the non-invasive case in early growth stages. In later stages, the invasive cells have degraded a signficant amount of extracellular matrix, leading to faster growth of the primary tumor than the non-invasive case. The final size of the tumor is determined by the volume of the growth-permitting regions. For invasive cases, at later growth stages, many invasive cells clump at the bounary of the growth-permitting region, which do not contribute to the tumor volume. Therefore, invasive tumors plateau at a smaller size than the non-invasive tumor.(EPS)

Figure S6
**Evolution of a simulated invasive tumor with cellular motility** 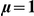 **in the heterogeneous ECM with a sinusoidal-like density distribution.** The mutation rate is 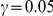 and ECM degradation ability is 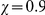. (a) Growing tumor on day 50. (b) Growing tumor on day 80. (c) Growing tumor on day 100. (d) Growing tumor on day 120. Note that the tumor morphology on days 80 and 120 are respectively shown in Figure 8(a) and (b) of the main paper.(TIF)

Figure S7
**Evolution of a simulated invasive tumor with cellular motility** 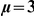 **in the heterogeneous ECM with a sinusoidal-like density distribution.** The mutation rate is 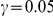 and ECM degradation ability is 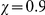. (a) Growing tumor on day 50. (b) Growing tumor on day 80. (c) Growing tumor on day 100. (d) Growing tumor on day 120. Note that the tumor morphology on days 80 and 120 are respectively shown in Figure 8(c) and (d) of the main paper.(TIF)
