# Supplementary figures and images for: Emergent Behaviors from a Cellular Automaton Model for Invasive Tumor Growth in Heterogeneous Microenvironments
Source: PLoS Comput Biol. 2011 Dec 22;7(12):e1002314. doi: 10.1371/journal.pcbi.1002314 (PMC3245298; doi:10.1371/journal.pcbi.1002314)

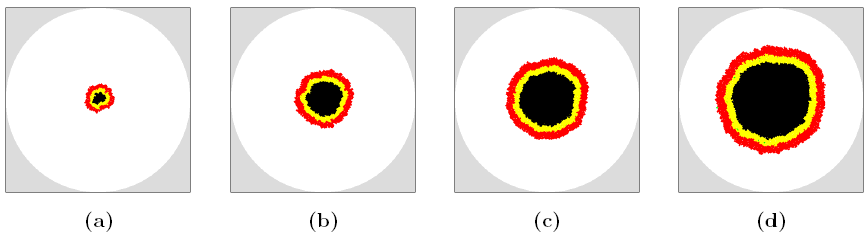

Supplement: Figure S1 — Evolution of a simulated non-invasive (proliferative) tumor in a homogeneous ECM. (a) Growing tumor on day 50. (b) Growing tumor on day 80. (c) Growing tumor on day 100. (d) Growing tumor on day 120. Note that the tumor morphology on day 100 is shown in Figure 5(a) of the main paper. (TIF) [file pcbi.1002314.s001.tif]

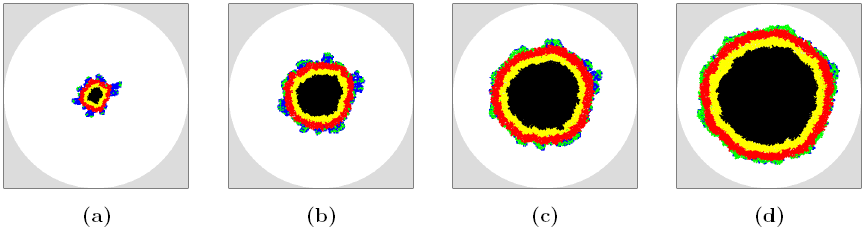

Supplement: Figure S2 — Evolution of a simulated invasive tumor with cellular motility in a homogeneous ECM. The mutation rate is and ECM degradation ability is . (a) Growing tumor on day 50. (b) Growing tumor on day 80. (c) Growing tumor on day 100. (d) Growing tumor on day 120. Note that the tumor morphology on day 100 is shown in Figure 5(b) of the main paper. (TIF) [file pcbi.1002314.s002.tif]

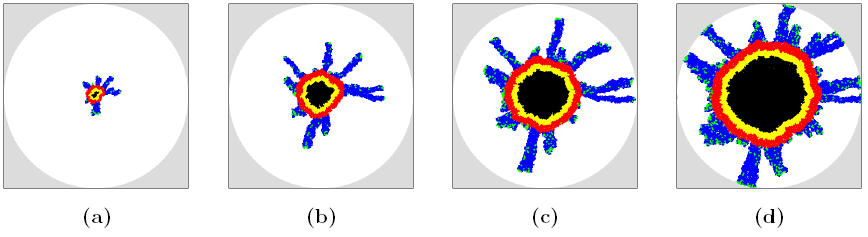

Supplement: Figure S3 — Evolution of a simulated invasive tumor with cellular motility in a homogeneous ECM. The mutation rate is and ECM degradation ability is . (a) Growing tumor on day 50. (b) Growing tumor on day 80. (c) Growing tumor on day 100. (d) Growing tumor on day 120. Note that the tumor morphology on day 100 is shown in Figure 5(c) of the main paper. (TIF) [file pcbi.1002314.s003.tif]

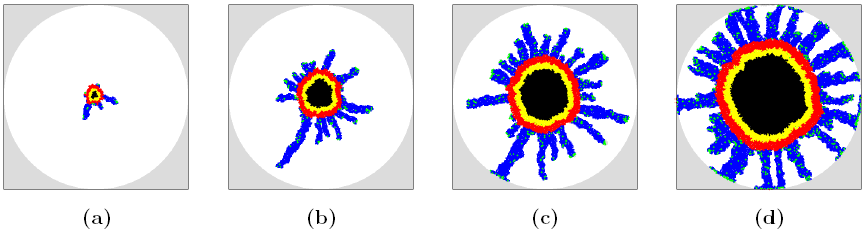

Supplement: Figure S4 — Evolution of a simulated invasive tumor with cellular motility in a homogeneous ECM. The mutation rate is and ECM degradation ability is . (a) Growing tumor on day 50. (b) Growing tumor on day 80. (c) Growing tumor on day 100. (d) Growing tumor on day 120. Note that the tumor morphology on day 100 is shown in Figure 5(d) of the main paper. (TIF) [file pcbi.1002314.s004.tif]

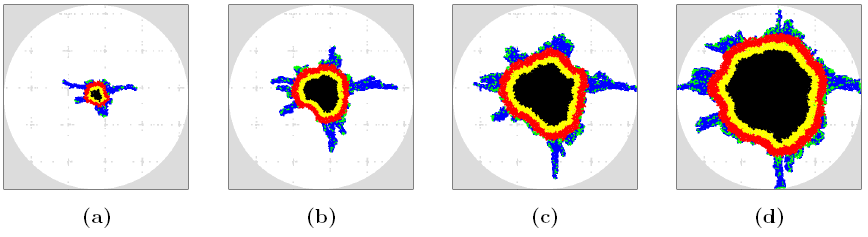

Supplement: Figure S6 — Evolution of a simulated invasive tumor with cellular motility in the heterogeneous ECM with a sinusoidal-like density distribution. The mutation rate is and ECM degradation ability is . (a) Growing tumor on day 50. (b) Growing tumor on day 80. (c) Growing tumor on day 100. (d) Growing tumor on day 120. Note that the tumor morphology on days 80 and 120 are respectively shown in Figure 8(a) and (b) of the main paper. (TIF) [file pcbi.1002314.s006.tif]

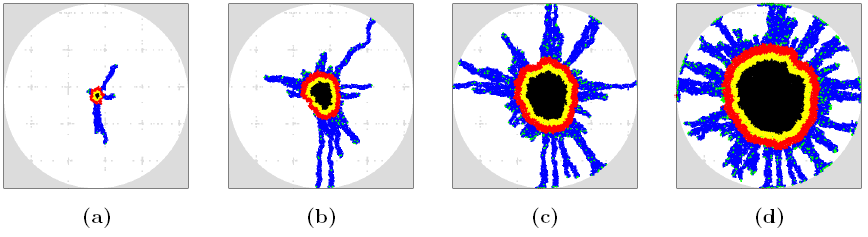

Supplement: Figure S7 — Evolution of a simulated invasive tumor with cellular motility in the heterogeneous ECM with a sinusoidal-like density distribution. The mutation rate is and ECM degradation ability is . (a) Growing tumor on day 50. (b) Growing tumor on day 80. (c) Growing tumor on day 100. (d) Growing tumor on day 120. Note that the tumor morphology on days 80 and 120 are respectively shown in Figure 8(c) and (d) of the main paper. (TIF) [file pcbi.1002314.s007.tif]
